# Decreased Myocardial 123I‐MIBG Uptake Without Sympathetic Denervation: An Autopsy Report of Autoimmune Autonomic Ganglionopathy

**DOI:** 10.1002/brb3.71388

**Published:** 2026-04-24

**Authors:** Daisuke Taniguchi, Kenya Nishioka, Yuta Ishiguro, Tsuyoshi Furuya, Takashi Ogawa, Kazunori Kajino, Yoshiaki Furukawa, Nobutaka Hattori

**Affiliations:** ^1^ Department of Neurology Juntendo University Graduate School of Medicine Bunkyo‐ku Tokyo Japan; ^2^ Department of Neurology Juntendo Tokyo Koto Geriatric Medical Center Bunkyo‐ku Tokyo Japan; ^3^ Department of Neurology Juntendo University Urayasu Hospital Chiba Japan; ^4^ Department of Pathology Juntendo Tokyo Koto Geriatric Medical Center Koto‐ku Tokyo Japan; ^5^ Neurodegenerative Disorders Collaborative Laboratory, RIKEN Center for Brain Science Wako Saitama Japan

## Abstract

**Introduction:**

Autoimmune autonomic ganglionopathy (AAG) is a rare disorder characterized by widespread autonomic failure due to antibodies targeting the ganglionic nicotinic acetylcholine receptor (gAChR). Although the clinical manifestations of AAG have been widely reported, autopsy findings in human cases remain undocumented.

**Methods:**

We examined the clinical course, imaging data, and autopsy findings of a patient with AAG who survived for 14 years. Clinical symptoms, cardiac MIBG scintigraphy results, treatment response, and histological features of sympathetic ganglia and cardiac nerve fibers were analyzed.

**Results:**

The patient initially showed markedly reduced MIBG cardiac uptake, which improved with intravenous immunoglobulin therapy. After a prolonged clinical course with recurrent infections and nutritional decline, he died at age 78. Autopsy revealed preserved thoracic sympathetic ganglia and intact postganglionic sympathetic fibers in the heart. Mild perivascular lymphocytic infiltration was observed, but no neuronophagia or dense inflammatory infiltrates. Phosphorylated α‐synuclein was absent in the central and peripheral nervous systems. The enteric plexus was preserved, without overt neuronal loss. Age‐associated tau pathology was confined to the entorhinal cortex.

**Conclusion:**

Despite functional sympathetic impairment evident in reduced MIBG uptake and severe gastrointestinal dysmotility, there was no histologic evidence of sympathetic nerve degeneration or neuronal loss in the enteric plexus, supporting a postsynaptic channelopathy mechanism in AAG. The mild inflammation observed may reflect systemic illness rather than primary autoimmune pathology. This is the first report correlating longitudinal clinical data, imaging, and postmortem pathology in AAG.

## Graphical abstract

Autopsy findings in autoimmune autonomic ganglionopathy (AAG) revealed preserved thoracic sympathetic ganglia, intact cardiac sympathetic fibers, and preserved enteric plexuses, providing pathological support for AAG as a channelopathy.

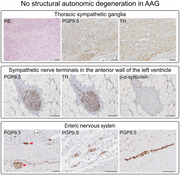

## 1 Introduction

Autoimmune autonomic ganglionopathy (AAG) is an acquired disorder of the autonomic nervous system caused by antibodies that bind the ganglionic neuronal nicotinic acetylcholine receptor (gAChR) (Vernino et al. 2000). Patients with AAG develop widespread autonomic failure and extra‐autonomic manifestations (Vernino et al. 2000; Nakane et al. 2020). Immunotherapy is effective and can provide long‐term disease control; however, disease recurrence is possible (Iodice et al. 2009). Metaiodobenzylguanidine (MIBG) scintigraphy is considered a useful tool for diagnosing AAG. In a study of 179 Japanese AAG patients with gAChR autoantibodies, 80% exhibited a decreased heart‐to‐mediastinum (H/M) MIBG uptake ratio in the early and/or delayed phases; after immunotherapy, the H/M ratio increased and autonomic symptoms improved (Nakane et al. 2020). This change in H/M ratio demonstrates that the pathologic autoantibodies induce a physiological block of ganglionic synaptic transmission. Previous pathological evaluations based on skin and sural nerve biopsies have reported loss of peripheral autonomic nerve fibers, raising the possibility that degenerative changes may also occur in AAG (Koay et al. 2021; Koike et al. 2013; Manganelli et al. 2011). However, to the best of our knowledge, neuropathologic findings in an affected patient have not been previously reported. We describe here the clinical course, imaging findings, and pathological findings at autopsy in a patient with AAG who lived for 14 years after diagnosis.

## 2 Clinical Summary

A previously healthy 64‐year‐old man presented with repeated syncope and other signs of autonomic dysfunction (hypohidrosis, frequent urination, constipation, and impotency). His pupils appeared enlarged on examination, with bilaterally sluggish light reflexes. Tilt table testing showed severe symptomatic orthostatic hypotension (BP 136/77, PR 71 bpm at a baseline angle of 0°; BP 78/41, PR 71 bpm at angle of 45°, 0 min; BP 58/33, PR 71 bpm at angle of 45°, 3 min), without an appropriate compensatory increase in heart rate, supporting impaired cardiac autonomic regulation. Serum immunoglobulin G (IgG) and IgM concentrations were slightly elevated and anti‐nuclear antibody testing was positive. Anti‐SS‐A/B antibody testing was negative. Cerebrospinal fluid testing showed normal cell numbers and protein concentration, but the IgG index was elevated (0.98). Electrocardiography showed a markedly reduced coefficient of variation in the R–R interval, measuring 0.51%, which is well below the age‐matched normal range (1.25%–2.68%), indicating severe cardiac autonomic dysfunction. MIBG scintigraphy showed decreased cardiac uptake and early and delayed H/M ratios of 1.97 and 1.71, respectively (normal value, >2.2); washout was not evaluated. Brain magnetic resonance imaging and whole‐body computed tomography were normal. No phosphorylated alpha‐synuclein deposition was detected in a skin biopsy. AAG was diagnosed based on an anti‐gAChR antibody concentration of 180 pmol/L (normal value, <50) and the clinical presentation of widespread autonomic failure.

Treatment of orthostatic hypotension using midodrine, amezinium methylsulfate, and fludrocortisone had minimal effect; intravenous methylprednisolone (1 g/day for 3 consecutive days) was effective but was discontinued owing to elevated liver enzymes. Intravenous immunoglobulin (0.4 g/kg/day for 5 consecutive days) was then administered, which improved his orthostatic hypotension and stopped the syncopal events. This therapy was repeated as needed based on symptom frequency, with a total of eight courses administered over the disease course. Post‐treatment scintigraphy showed improved cardiac MIBG uptake with early and delayed H/M ratios of 4.40 and 5.18, respectively; the washout rate was 19.7%.

Ten years later, at the age of 74 years, the patient was experiencing syncope every day. Intravenous immunoglobulin therapy was no longer effective, and he was malnourished because of impaired intestinal peristalsis. Total parenteral nutrition was eventually required after his general condition had gradually deteriorated. He developed repeated cases of aspiration pneumonia and urinary tract infections and died at the age of 78.

## 3 Pathological Findings

**FIGURE 1:**
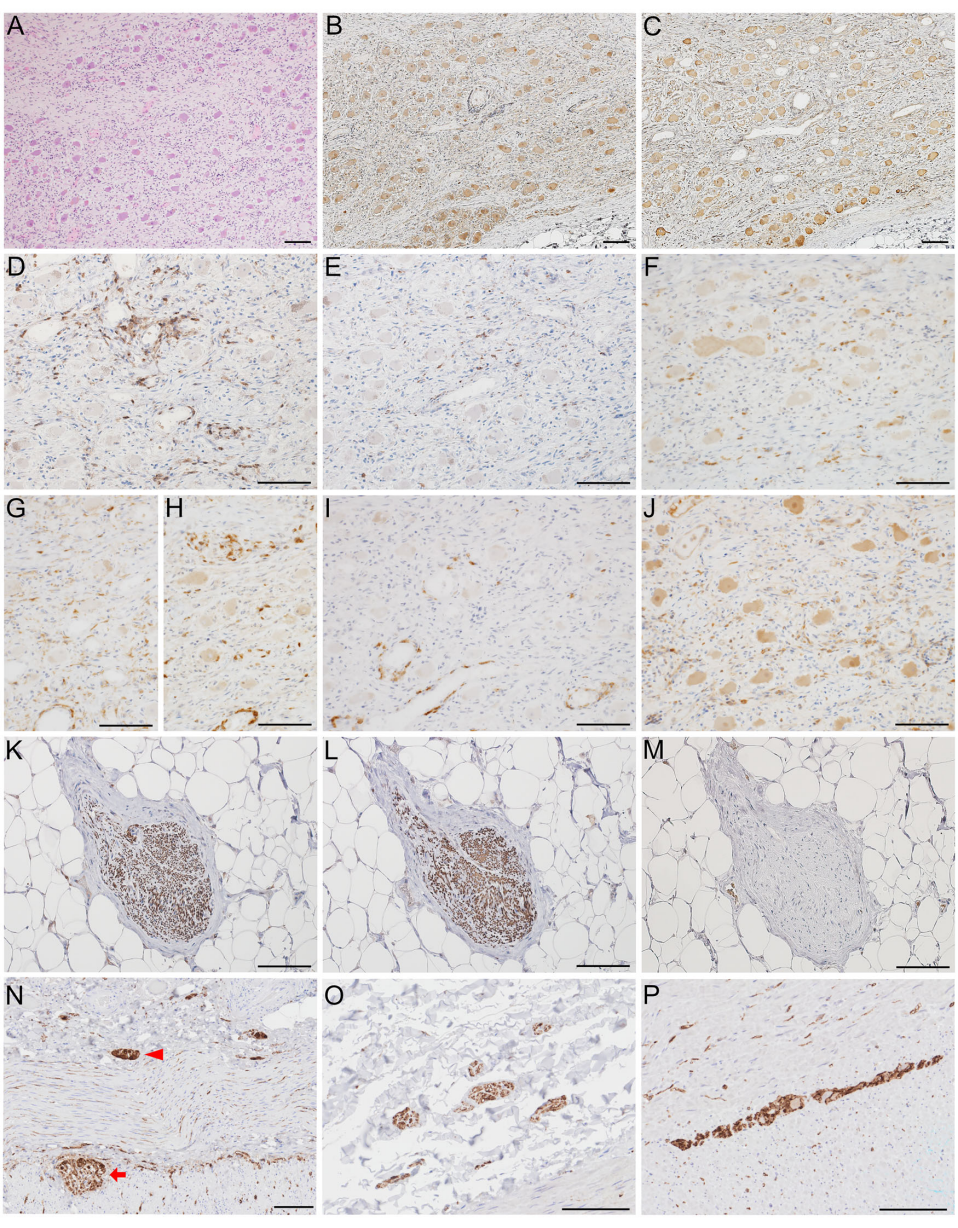
Neuropathological findings in thoracic sympathetic ganglia: hematoxylin and eosin (A), protein gene product 9.5 (B), tyrosine hydroxylase (C), CD45 (D), CD68 (E), CD3 (F), CD4 (G), CD8 (H), CD20 (I), and immunoglobulin G (J). Findings in sympathetic nerve terminals in the anterior wall of the left ventricle: protein gene product 9.5 (K), tyrosine hydroxylase (L), and phosphorylated alpha‐synuclein (M). Enteric nervous system identified by PGP9.5 immunostaining: ileum showing the submucosal (Meissner) plexus (red arrowhead) and the myenteric (Auerbach) plexus (red arrow) (N); submucosal plexus in the colon (O); and myenteric plexus in the colon (P). Scale bars: 100 µm.

Autopsy revealed hemorrhagic pneumonia and diffuse alveolar damage. No evidence of occult malignancy was identified. No gross dilatation of the gastrointestinal tract or urinary bladder was observed. Thoracic sympathetic ganglion cells were preserved (Figure 1A) and stained positive for antibodies to protein gene product 9.5 (PGP9.5) and tyrosine hydroxylase (Figure 1B,C). CD45+ lymphocytes and CD68+ macrophages were detected in connective tissue (Figure 1D,E). CD3+ T‐cells consisted of both CD4+ and CD8+ cells (Figure 1F,H). CD20+ B‐cells were scattered; some surrounded vessel walls (Figure 1I). IgG‐positive cells were detected in connective tissue, but not in ganglion cells (Figure 1J). Sympathetic nerve bundles in the anterior wall of the left ventricle were well preserved and stained positive for both PGP9.5 and tyrosine hydroxylase (Figure 1K,L). Phosphorylated alpha‐synuclein pathology was not detected in either the peripheral or central nervous systems (Figure 1M). In the ileum, both the submucosal (Meissner) plexus (red arrowhead, Figure 1N) and the myenteric (Auerbach) plexus (red arrow, Figure 1N) were identified by immunostaining for PGP9.5. Similarly, in the colon, the submucosal plexus (Figure 1O) and the myenteric plexus (Figure 1P) were also identified by PGP9.5 immunostaining. Age‐associated neurofibrillary tangles and argyrophilic grains were present in the entorhinal cortex.

## 4 Discussion

In the AAG patient presented here, histopathological analysis at autopsy showed no postganglionic sympathetic nerve degeneration from the sympathetic ganglion cells to the distal nerve bundles in the epicardium, even though initial MIBG scintigraphy showed low cardiac uptake and decreased H/M ratio, which improved after immunotherapy. An animal model of AAG confirmed that the disorder is a postsynaptic channelopathy (Lennon et al. 2003). Thus, autoantibody‐induced impairment at the sympathetic ganglia could lead to dysfunction of noradrenergic transporters at postganglionic sympathetic nerve endings, resulting in decreased MIBG uptake.

Lymphocytes were observed in the connective tissue and around vessel walls in thoracic sympathetic ganglia; however, no obvious neuronophagia or clear lymphocytic infiltration was observed. We do not know whether the observed accumulation of immune cells in the sympathetic ganglia was caused by the patient's AAG or was related to systemic inflammation caused by infection. Inflammatory changes have not been reported in AAG animal models (Lennon et al. 2003; Yamakawa et al. 2022).

Experimental models of autoimmune autonomic ganglionopathy have shown gastrointestinal and bladder dilatation accompanied by neuronal loss in the enteric plexus (Lennon et al. 2003; Vernino et al. 2004, 2003). In contrast, in the present case, the enteric plexus was identifiable without obvious neuronal loss. Although peripheral autonomic fiber loss has been reported in human AAG, our autopsy findings demonstrate preservation of the autonomic ganglia and the enteric nervous system, providing pathological support for AAG as a channelopathy.

## Author Contributions

**Daisuke Taniguchi**: conceptualization, investigation, writing – original draft, funding acquisition. **Kenya Nishioka**: conceptualization, writing – review and editing, supervision, investigation. **Yuta Ishiguro**: investigation, writing – review and editing. **Tsuyoshi Furuya**: writing – review and editing, investigation. **Takashi Ogawa**: investigation, writing – review and editing. **Kazunori Kajino**: investigation, writing – review and editing, validation. **Yoshiaki Furukawa**: writing – review and editing, supervision. **Nobutaka Hattori**: supervision, writing – review and editing.

## Funding

The authors have nothing to report.

## Ethics Statement

This study was conducted in accordance with the Declaration of Helsinki. Ethical approval for this case report was obtained from the Institutional Review Board of Juntendo University (Approval No. M19‐0235). Written informed consent for autopsy and publication was obtained from the patient's next of kin.

## Data Availability

The data that support the findings of this study are available from the corresponding author upon reasonable request.
